# Circulating ERBB3 levels are inversely associated with the risk of overweight-related hypertension: a cross-sectional study

**DOI:** 10.1186/s12902-021-00793-8

**Published:** 2021-06-27

**Authors:** Lijun Zhu, Zhengmei Fang, Yuelong Jin, Weiwei Chang, Mengyun Huang, Yan Chen, Yingshui Yao

**Affiliations:** 1 Department of Epidemiology and Biostatistics, School of Public Health, Wannan Medical College, No. 22, Wenchang Road, Anhui 241002 Wuhu, China; 2 Institute of Chronic Disease Prevention and Control,Wannan Medical College, No. 22, Wenchang Road, Anhui 241002 Wuhu, China; 3 Department of Medicine, Anhui College of Traditional Chinese Medicine, No.18, Wuxia Shanxi Road, Anhui 241003 Wuhu, China

**Keywords:** ERBB3, Hypertension, Overweight, Body mass index

## Abstract

**Background:**

Hypertension and overweight are independent risk factors for cardiovascular disease, and overweight increase the risk of developing high blood pressure. ERBB3( also known as HER3) plays a considerable role in the development of cardiovascular diseases. However, the effect of ERBB3 levels in hypertensive overweight patients is unknown. The aim of this study was to assess the association between ERBB3 levels and hypertension in overweight Chinese patients.

**Methods:**

We evaluated the height,weight, blood pressure, biochemical indicators, and ERBB3 levels in 128 Chinese adults aged 33–79 years. Plasma ERBB3 levels were assessed by the enzyme-linked immunosorbent assay, and body mass index(BMI) was calculated as body weight divided by height squared. Participants were allocated into three groups according to blood pressure and BMI: healthy control (CNT, *n* = 31; normotensive and non-overweight), hypertension (HT, *n* = 33; hypertension and non-overweight), and hypertension with overweight (HTO, *n* = 64; hypertension and overweight). Statistical significance was defined as a two-tailed *P* < 0.05.

**Results:**

There was no significant difference in mean ERBB3 levels among the three groups, although a linear decrease from CNT (1.13 ± 0.36), HT (1.03 ± 0.36), to HTO (0.84 ± 0.26 ng/mL) was observed in men (*P* = 0.007). Among the drinking population, the ERBB3 level was significantly reduced in the HTO group as compared with those of the CNT and HT groups (0.76 ± 0.23 versus 1.18 ± 0.37 and 1.20 ± 0.30, respectively). ERBB3 levels were negatively correlated with diastolic blood pressure in men (*r*= − 0.293, *P* = 0.012), smoking (*r*= − 0.47, *P* = 0.004), and drinking (*r* = − 0.387, *P* = 0.008). BMI in men and among drinkers, and uric acid among drinkers were negatively correlated with ERBB3 levels. Multivariate conditional logistic regression showed that plasma ERBB3 levels were associated with a reduced risk of HTO in men [odds ratio (OR) 0.054; 95 % confidence interval (*CI)*: 0.007–0.412) and drinkers (*OR* 0.002; 95 % *CI*: 0.000–0.101).

**Conclusions:**

ERBB3 may contribute to the pathogenesis of hypertension in overweight patients, with BMI, gender, and drinking all potentially modulating the process.

## Background

Hypertension is a condition in which the blood vessels have persistently raised pressure,and is one of the most pressing public health challenges. The global burden of hypertension has been growing in recent decades, largely driven by population growth, changes in lifestyle, and aging [1]. From 1975 to 2015, the number of adults with high blood pressure increased from 594 million to 1.13 billion, with a particularly large increase in low-income and middle-income countries[2]. Based on data from 451,755 Chinese individuals, the prevalence of hypertension among adults was 27.9 %, and was similar among men (28.6 %) and women (27.2 %) [3]. Previous studies have shown that the prevalence of hypertension may be accounted for by increasing body mass index (BMI) [4]. Indeed, a high BMI (i.e., overweight or obese) is a major risk factor for several chronic diseases, including diabetes, cardiovascular diseases, and hypertension [5]. Epidemiological studies have shown an association between BMI and blood pressure both normal-weight and overweight patients [6]. Previous studies have reported that BMI is strongly associated with hypertension in northern Chinese adults [7], and could explain 45 % of the age-adjusted increase in diastolic blood pressure (DBP) over the study period in an Indians population [8].

The ERBB (also known as HER) family of membrane-bound tyrosine kinase receptors comprises ERBB1, ERBB2, ERBB3, and ERBB4, which activate potent signalling pathways that mediate cell proliferation or differentiation[9]. Unlike other ERBB family members, the function of ERBB3 (HER3) has been investigated less frequently because its intracellular kinase domain is thought to be an inactive pseudokinase, thereby relying on interactions with other ERBB partners [10]. To date, ERBB3 has been widely used as a tumour marker; however, it was recently reported to be associated with cardiovascular diseases [11].

*ERBB3* mRNA expression levels on the surface of monocytes were reported to be inversely correlated with tumor necrosis factor alpha in subjects with heart failure but not in human subjects without heart failure[12]. In addition, the brown fat–enriched secreted factor NRG4 has been proposed as a potential target to treat obesity-associated disorders, which primarily signals through ERBB3 to regulate diverse biological processes [13]. Emerging evidence suggests that dysregulation of ERBB3 is important in mediating hyperglycaemia-induced vascular dysfunction [14]. A case-control study showed that *ERBB3* genetic polymorphisms are associated with the pathogenesis of coronary artery disease [15]. Moreover, recent animal studies showed that transient receptor potential vanilloid 4 (TRPV4) ion channels, a major Ca^2+^ influx pathway in endothelial cells, contributes to obesity-induced hypertension [16]. Moreover, G protein-coupled receptors regulate TRPV4 activity in the vasculature by mediating ERBB family transactivation [17]. Thus, we hypothesized that the ERBB3 may participate in the process that leads to the occurrence and development of hypertension in the and overweight context.

Given that ERBB3 may play a considerable role in the development of cardiovascular diseases, the aim of this study was to assess the relationship between plasma ERBB3 levels and hypertension in overweight Chinese adults, and to provide the basis for the pathogenesis of overweight-related hypertension.

## Methods

### Study Subjects

The subjects were recruited via the Health Management Center and Physical Examination Center at Yijishan Hospital of Wannan Medical College from July 2019 to August 2019. According to the ‘Seventh report of the joint national committee on prevention, detection, evaluation, and treatment of high blood pressure’, hypertension is generally defined as a systolic blood pressure (SBP) of ≥ 140 mmHg or a DBP of ≥ 90 mmHg or the use of antihypertensive medications. For adults, BMI China reference standard defines overweight as a BMI ≥ 24. Accordingly, in this study, the healthy control group (CNT) was defined as both normotensive and BMI < 24, the hypertension group (HT) was defined as both hypertensive and BMI < 24, the hypertension with overweight group (HTO) was defined as both hypertension and BMI ≥ 24. Finally, 128 adults (aged 33–79 years), including 31 in the CNT group, 33 in the HT group, and 64 in the HTO group, were included in this study for assessing the association between plasma ERBB3 levels and hypertension with in the context of overweight.

The study was approved by the Ethics Committee of the First Affiliated Yijishan Hospital of Wannan Medical College (Wuhu, China). Written informed consent was obtained from all participants.

### Data collection

At physical examination, all subjects were measured for height and weight, and blood pressure. BMI was calculated as body weight (kg) divided by height squared (m^2^). A well-trained research staff member measured blood pressure once using an electronic sphygmomanometer with the participant in the sitting position after at least 5 min of rest. Subjects were classified as smokers (including current and ex-smokers) or non-smokers, and as drinkers (including current and ex-drinkers) or non-drinkers.

### Biochemical analyses

Blood samples were obtained from the subjects after an overnight fast for at least 10 h. Total cholesterol (TC), triglyceride (TG), high-density lipoprotein cholesterol (HDL-C), low-density lipoprotein cholesterol (LDL-C), glucose (GLU), and uric acid (UA) were measured using standard methods at the physical examination institution.

### Enzyme-linked immunosorbent assay (ELISA)

Three millilitre venous blood samples were collected and centrifuged at 3000 rpm for 10 min. The supernatant was collected using a micropipette and stored in at − 80 °C until use. The plasma samples were then thawed at room temperature for ELISA using a commercial kit ( CUSABIO,Catalogue No.: No. CSB-EL007765HU) according to the manufacturer’s instructions. All measurements were repeated twice to calculate the average of parallel samples.

### Statistical analysis

Statistical analysis was performed using SPSS software (version 18.0, SPSS Inc., Chicago, IL, USA). The normality assumption of the data was tested using the Kolmogorov-Smirnov test. Quantitative data are expressed as mean ± standard deviation (SD) and categorical data are expressed as frequencies (*n*, %). The differences in variables among the groups were determined by one-way analysis of variance (ANOVA) or the chi-square test. Pearson’s correlation coefficient was used for assessing correlations of normally distributed data. Multiple unconditional logistic regression analysis with effect sizes [odds ratios (*ORs*) and 95 % confidence intervals (*CIs*)] was applied to estimate the risk of hypertension with and without overweight after adjusting for potential confounding factors. Statistical significance was defined as a two-tailed *P* < 0.05.

## Results

### Characteristics of the study participants

As shown in Table 1, there were no differences in age; TC, LDL-C, HDL-C,and GLU levels; or the distribution of sex and drinking status among the three groups (*P* > 0.05). The TG level was significantly higher in the HTO group than that in the CNT and HT group. The UA levels in the HTO group were higher than those in the HT group.

**Table 1:** Characteristics of the study participants among CNT, HT and HTO groups

Characteristic	Group	CNT (*n* = 31)	HT(*n* = 33)	HTO (*n* = 64)	*F/χ*^*2*^	*P*
Gender	Male	18(58.1)	22(66.7)	32(50.0)	2.512	0.285
	Female	13(41.9)	11(33.3)	32(50.0)		
Age (year)		60.32±5.82	63.67±8.35	62.61±8.69	1.476	0.232
Blood pressure(mmHg)	SBP	122.84±13.27	148.94±15.86*	147.23±14.26*	35.19	<0.001
	DBP	74.29±9.58	83.00±9.62*	83.59±10.97*	9.18	<0.001
TC(mmol/L)		4.86±0.90	4.44±0.82	4.51±0.97	1.961	0.145
TG(mmol/L)		1.61±0.78	1.43±0.58	2.03±1.10*#	5.354	0.006
HDL-C(mmol/L)		1.53±0.36	1.51±0.38	1.40±0.24	2.414	0.094
LDL-C(mmol/L)		2.59±0.73	2.28±0.68	2.18±0.82	2.966	0.055
UA(umol/L)		360.01±88.18	329.27±80.75	385.63±95.21#	4.327	0.015
GLU(mmol/L)		5.08±0.95	5.45±0.94	5.45±1.14	1.430	0.243
BMI(kg/m^2^)		21.43±1.73	21.40±1.90	26.71±2.51*#	91.753	<0.001
ERBB3(ng/ml)		1.00±0.43	1.01±0.35	0.92±0.37	0.792	0.455
Current smoker	Yes	12(38.7)	14(42.4)	10(15.6)	10.003	0.007
	No	19(61.3)	19(57.6)	54(84.4)		
Current drinker	Yes	12(38.7)	12(36.4)	22(34.4)	0.174	0.917
	No	19(61.3)	21(63.6)	42(65.6)		

*CNT* healthy control group, *HT* hypertension group, *HTO* hypertension with overweight group, *BMI* body mass index, *SBP* systolic blood pressure, *DBP* diastolic blood pressure, *GLU* glucose, *TC* total cholesterol, *TG* triglycerides, *HDL-C* high-density lipoprotein cholesterol, *LDL-C* low-density lipoprotein cholesterol, *UA* uric acid. P: All participants had the variables analyzed by one-way ANOVA or Chi- square test among CNT, HT and HTO groups.*: *p* < 0.05 vs. CNT.# : *p* < 0.05 vs. HT

### Comparison of plasma levels of ERBB3 among the three groups

There were no statistically significant differences in ERBB3 levels among the three groups. By gender subgroup, the plasma concentrations of ERBB3 showed a linear decrease in the CNT, HT and HTO groups in men (1.13 ± 0.36, 1.03 ± 0.36, and 0.84 ± 0.26 ng/mL, respectively; *P* = 0.007). In the subgroup of alcohol drinkers, the ERBB3 level was significantly decreased in the HTO group as compared with those of the CNT and HT groups (0.76 ± 0.23 versus 1.18 ± 0.37 and 1.20 ± 0.30, respectively) (Table 2).

**Table 2:** Stratification analyses for comparison plasma levels of ERBB3 among the three groups

Characteristic	Group	CNT (*n* = 31)	HT (*n* = 33)	HTO (*n* = 64)	*F*	*P*
Total population		1.00±0.43	1.01±0.35	0.92±0.37	0.792	0.455
Gender	Male	1.13±0.36	1.03±0.36	0.84±0.26*#	5.265	0.007
	Female	0.82±0.47	0.97±0.34	1.00±0.43	0.816	0.448
Current smoker	Yes	1.20±0.41	1.06±0.40	0.89±0.30	1.770	0.186
	No	0.88±0.40	0.97±0.31	0.93±0.38	0.284	0.753
Current drinker	Yes	1.18±0.37	1.20±0.30	0.76±0.23*#	12.483	<0.001
	No	0.89±0.44	0.90±0.33	1.00±0.40	0.810	0.449

*CNT* healthy control group, *HT* hypertension group, *HTO* hypertension with overweight group. P: All participants had the variables analyzed by one-way ANOVA or Chi- square test among CNT, HT and HTO groups.*: *p* < 0.05 vs. CNT.# : *p* < 0.05 vs. HT.*: *p* < 0.05 vs. Control.# : *p* < 0.05 vs. hypertension

### Correlation of plasma ERBB3 levels and clinical characteristics

Table 3 summarises the Pearson correlation coefficients between ERBB3 levels and hypertension risk factors stratified by sex, smoking, and drinking. ERBB3 levels were negatively correlated with DBP in men (*r* = − 0.293, *P* = 0.012), smoking (*r* = − 0.47, *P* = 0.004), and drinking (*r* = − 0.387, *P* = 0.008). Notably, BMI also correlated negatively with ERBB3 levels among men and drinkers. Similarly, UA levels were negatively correlated with ERBB3 levels among drinkers (all *P* < 0.05).

**Table 3:** Correlation of plasma ERBB3 levels and clinical characteristics by gender, smoking, and drinking

Characteristic	Male		Female		Nonsmoking		Smoking		Nondrinking		Drinking		Total population	
	*r*	*P*	*r*	*P*	*r*	*P*	*r*	*P*	*r*	*P*	*r*	*P*	*r*	*P*
SBP(mmHg)	-0.155	0.193	0.038	0.779	0.019	0.855	-0.194	0.256	0.022	0.846	-0.239	0.109	-0.062	0.49
DBP(mmHg)	-0.293	0.012	0.019	0.89	-0.011	0.914	-0.47	0.004	-0.03	0.788	-0.387	0.008	-0.129	0.146
TC(mmol/L)	0.07	0.562	-0.094	0.491	0.048	0.647	-0.053	0.76	-0.05	0.655	0.071	0.637	-0.003	0.971
TG(mmol/L)	0.014	0.907	0.138	0.31	0.149	0.157	-0.11	0.521	0.117	0.293	-0.001	0.995	0.079	0.376
HDL-C(mmol/L)	0.031	0.795	0.03	0.826	0.045	0.669	0.113	0.511	0.044	0.691	-0.009	0.953	0.023	0.794
LDL-C(mmol/L)	0.068	0.57	-0.202	0.136	-0.054	0.612	-0.059	0.733	-0.151	0.176	0.088	0.563	-0.057	0.526
Glu(mmol/L)	0.034	0.776	0.117	0.39	0.156	0.138	-0.095	0.582	0.015	0.894	0.214	0.153	0.077	0.391
UA(umol/L)	-0.169	0.156	0.081	0.551	-0.047	0.66	-0.075	0.663	0.08	0.475	-0.375	0.01	-0.041	0.647
BMI(kg/m2)	-0.252	0.034	0.089	0.516	-0.017	0.872	-0.173	0.321	0.065	0.561	-0.389	0.008	-0.079	0.378

*CNT* healthy control group, *HT* hypertension group, *HTO* hypertension with overweight group, *BMI* body mass index, *SBP *systolic blood pressure, *DBP* diastolic blood pressure, *GLU *glucose, *TC* total cholesterol, *TG* triglycerides, *HDL-C* high-density lipoprotein cholesterol, *LDL-C *low-density lipoprotein cholesterol, *UA *uric acid

### Multinomial logistic regression

Table 4 shows the associations of HT and HTO with increasing levels of ERBB3 within subgroups of sex, current smoking, and drinking. Plasma ERBB3 levels were negatively associated with hypertension and overweight among men (*OR* 0.054, 95 % *CI* 0.007–0.412) and drinking (*OR* 0.002, 95 % *CI* 0.000–0.101). After adjusting for age and sex, the association remained significant.

**Table 4:** Multinomial logistic regression analysis between ERBB3 and HT and HTO by gender, smoking, and drinking

Characteristic	Group	Mode1		Mode2	
		HT(n=33)	HTO(n=64)	HT(n=33)	HTO(n=64)
Gender	Male	0.414(0.064-2.685)	0.054(0.007-0.412)	0.401(0.060-2.707)	0.059(0.008-0.437)
	Female	3.007(0.305-29.645)	3.612(0.495-26.354)	2.589(0.274-24.448)	2.997(0.426-21.077)
Current smoker	Yes	0.379(0.046-3.146)	0.096(0.007-1.250)	0.368(0.043-3.179)	0.100(0.008-1.284)
	No	2.006(0.332-12.113)	1.503(0.315-7.180)	1.843(0.305-11.139)	1.433(0.315-6.527)
Current drinker	Yes	1.221(0.106-14.070)	0.002(0.000-0.101)	1.354(0.108-16.981)	0.002(0.000-0.111)
	No	1.077(0.177-6.537)	2.242(0.481-10.458)	0.877(0.133-5.784)	2.036(0.439-9.436)

Mode1-no adjustment;Mode2- adjusted for age, gender

## Discussion

The Framingham Study showed that hypertension [18] and overweight [19] are both independent risk factors for cardiovascular disease, and approximately 30 % of hypertensive individuals can be classified as obese [20]. In addition, ERBB3 has been reported to play an important role in the maintenance and development of cardiovascular disorders [11, 15]. This study is the first to demonstrate the importance of ERBB3 levels in overweight patients with hypertension. We observed a negative association between ERBB3 levels and hypertension with overweight among only men and drinkers. These results indicate that ERBB3 levels might contribute to the development of hypertension in overweight individuals.

A previous animal study demonstrated that ERBB3 receptors might be involved in blood pressure regulation through NRG-1/ERBB signalling as an antihypertensive system, but that this effect is probably not that strong [21]. In this study, we observed a negative correlation between ERBB3 and DBP in the male, drinking and smoking groups. A previous reports suggested that males are generally at greater risk for hypertension than age-matched females [22]. Another study reported that environmental exposure to drinking and smoking was associated with adult hypertension in the Japanese population [23]. Remarkably, DBP showed increased sensitivity to environmental changes, and more strongly predicted cardiovascular disease risk in younger Chinese adults [24]. In the present study, BMI (both in men and in the drinking population) and UA (among drinkers) correlated negatively with ERBB3 levels. Other studies found men are more frequently overweight, and consume more alcohol than women [25]. Additionally, genetic studies have shown that *ERBB3* is responsible for variations in the LDL-C serum concentration, and plays a role in lipid homeostasis and obesity [26]. Taken together, BMI, gender, and drinking all potentially modulate the association between ERBB3 levels and hypertension in overweight individuals.

However, the mechanisms that link ERBB3 and hypertension and overweight are currently unclear. Previous studies have explored potential mechanisms linking adiposity and high blood pressure, including sympathetic nervous system activation, activation of the renin–angiotensin system, inflammatory responses, and insulin resistance [27]. It may be speculated that ERBB3 participates in several networks related to lipid metabolism which is a major variable in the aetiology of both overweight and hypertension [28]. In addition, ERBB3 participates in neutrophil survival and ERBB3 inhibitors play positive roles in accelerating inflammation resolution [29], which is a known predisposing factor for the development and progression of hypertension and overweight. Signalling interactions have been reported between ERBB3 family members and insulin-like growth factor-1 receptor (IGF-IR) [30]. Our group previously reported that IGF-IR may contribute to the genetic susceptibility to hypertension [31], and higher *IGF-IR* mRNA expression levels were observed in obese children [32]. Thus, it is reasonable to consider that the plasma ERBB3 levels might play an important role in the regulation of hypertension in overweight by affecting IGF-IR.

There are a few limitations to our study. Firstly, the potential bias in cross-sectional studies can often distort the results of epidemiological associations. Secondly, no significant differences in plasma ERBB3 levels were observed among the three groups in the total population, despite the fact that strict standards were used to select representative cases and controls. The relatively small sample size may have led to weak statistical power. Thus, further prospective studies are required to confirm these observations and to increase the sample size. In particular, more research involving the obese population is warranted.

## Conclusions

In summary, our results provide the first demonstration that the expression of ERBB3 was significantly downregulated in overweight individuals with hypertension, and that BMI, gender, and drinking all potentially modulate the process. Further larger studies may help to elucidate the relationship and role of ERBB3 levels in the pathogenesis of hypertension in overweight individuals.

## Data Availability

The datasets during and/or analyzed during the current study will be available from the corresponding author on reasonable request.

## Abbreviations

CNT: Healthy control group
HT: Hypertension group
HTO: Hypertension with overweight group
BMI: Body mass index
SBP: Systolic blood pressure
DBP: Diastolic blood pressure
ELISA: Enzyme-linked immunosorbent assay
GLU: Glucose
TC: Total cholesterol
TG: Triglycerides
HDL-C: High-density lipoprotein cholesterol
LDL-C: Low-density lipoprotein cholesterol
UA: Uric acid
